# Enrichment-induced behavioural changes in MPTP mice

**DOI:** 10.6026/973206300211672

**Published:** 2025-06-30

**Authors:** Geetha S., Vinutha Shankar M.S

**Affiliations:** 1 Department of Physiology, Sri Devaraj Urs Medical College, Kolar, Karnataka, India

**Keywords:** Parkinson's disease (PD), enriched environment, 1-methyl-4- phenyl 1, 2, 3, 6-tetrahydropyridine (MPTP) mice

## Abstract

Parkinson's disease (PD) is a progressive neurodegenerative disorder characterized by motor and non-motor deficits due to dopaminergic neuron loss. Neuro-protective effects of enriched environment have been well established. Therefore, it is of interest to examine the effects of enriched housing in a 1-methyl-4- phenyl 1, 2, 3, 6-tetrahydropyridine (MPTP)-induced mouse model of PD. Thirty-two male C57BL/6 mice were divided into standard and enriched housing groups, with MPTP or saline treatment. MPTP mice in standard housing showed impaired motor function, whereas MPTP mice in enriched housing did not show decline in motor function, demonstrating the beneficial effects of environmental enrichment in MPTP mice.

## Background:

Parkinson's disease (PD) is the second most common age-related neurodegenerative disease [1]. PD is a progressive neurodegenerative disorder characterized by loss of dopaminergic neurons in substantia nigra pars compacta (SNpc), leading to motor and non-motor symptoms [2]. It has a global prevalence of more than 6 million individuals [3], affecting nearly 3% individuals aged 65 and older [4, 5]. Multiple factors like, increasing life expectancy, increasing industrialization and declining smoking rates, could increase disease burden globally, which highlight its significant economic, social and public health impact [6, 7]. This rising disease incidence and prevalence globally make it a disease with huge economic, social and public health importance [8]. Pharmacological and surgical interventions offer only symptomatic relief and not a definitive cure for the disease. Hence, there is increasing interest in non-pharmacological strategies such as environmental enrichment as potential therapeutic approaches, aimed at enhancing neuro-protection and functional recovery. Consequently, researchers have developed animal models to replicate key features of PD, enabling a deeper understanding of its pathophysiology and the exploration of potential therapeutic interventions [9, 10]. Few commonly used neurotoxins to develop the PD models by inducing neuro-degeneration in animal brains are paraquat, rotenone, 6-hydroxydopamine (6 OHDA), and 1-methyl-4- phenyl 1, 2, 3, 6-tetrahydropyridine (MPTP) [11]. For decades, MPTP mouse model is being extensively used to study pathophysiology of PD and test potential intervention, due to their practicality, cost-effectiveness and stronger clinical relevance compared to other toxin-based models [12]. Sensitivity of mice strains to MPTP varies. Different strains or even the same strain from a different source shows varying sensitivity to MPTP in terms of loss of DA neurons in SNpc and striatum. C57BL/6 mice are most sensitive to MPTP intoxication [13, 14]. Numerous studies have demonstrated engaging in physical activity or exercise can alleviate motor symptoms of PD and improve patients' quality of life [15, 16- 17]. Environmental enrichment (EE) is a housing condition for study animals that involves providing a stimulating environment with increased physical and social activity, by increasing the number of cagemates, providing various toys like ladders, balls, different textured papers, running wheels *etc.* [18]. In preclinical models, EE has been shown have beneficial effects on various neurodegenerative disorders [19]. It has been demonstrated that EE offers potential benefits including increased neurogenesis, and improved motor function [20]. Researchers have also demonstrated that social enrichment, a component of EE, attenuates nigrostriatal lesioning and reverses motor impairments in a progressive MPTP mouse model of PD [21]. However, EE effects in alleviating motor deficits in PD models have been inconsistent across studies due to variations in EE paradigms, disease models, and behavioural assessments. Therefore, it is of interest to show the enriched housing influences of motor function in the MPTP mouse model of Parkinson's disease.

## Materials and Methods:

## Animals and housing:

Thirty-two male C57BL/6 mice (8 - 10 weeks old, 22-24g) were procured from a licensed supplier and housed under standard laboratory conditions (12-hour light/dark cycle, controlled temperature and humidity) with ad libitum access to food and water. Experimental design: Following a week of acclimatization, mice were trained for behavioural tests and randomly divided into standard housing (SH, n=16) and enriched environment (EE, n=16). Each group was subdivided into MPTP-treated (n=8) and saline-treated (control, n=8) subgroups. EE comprised large cages with tunnels, ladders, nesting material and toys and housed 8 mice each. SH cages were standard lab mice cages and contained 2 mice each. Seven days after the last injection, behavioural assessments were performed using Rotarod test, Actophotometer Test and the Hanging test. MPTP administration: PD was induced by intraperitoneal injections of MPTP hydrochloride (30 mg/kg/day) for five consecutive days (sub-acute regimen). Control mice received equal volume of saline injections.

## Behavioural assessments:

To assess the motor deficits associated with Parkinson's disease and the influence of environmental enrichment, mice were subjected to four motor function tests: Rotarod, Actophotometer, Hang test and Narrow beam walk test. Rotarod test: Motor coordination and balance were assessed using a constant speed rotarod apparatus. The apparatus consists of a horizontal rotating rod that spins at a steady speed, on which the animal must balance. Rod is divided into multiple sections for simultaneous testing of multiple animals. Apparatus contains a sensor which detects when an animal falls off the rod, recording latency before the fall. Before MPTP injection, mice were trained on rotarod at a speed of 10rpm for 10 min once a day for 5 consecutive days. Same was repeated for the tests, following the last injection of MPTP. Tests were repeated thrice at an interval of 5 minutes between tests. Time spent by the animal on the rotating rod was recorded, and mean latencies were calculated and compared [22].

## Acto-photometer test:

Locomotor activity was measured by recording number of beam breaks. Animals were tested for 5 minutes. Apparatus consists of photoelectric cells coupled in a circuit with a counter. Light beam striking photoelectric cell completes the circuit. An animal was placed in the metal box and when it walked through it, lights of photocell were being cut off and a count was recorded. All mice were initially placed inside the apparatus for five minutes to acclimatize. During testing, mice were kept for five minutes in the apparatus and readings were recorded as activity scores [23].

## Hang test:

Muscular strength (grip strength) and endurance were assessed by measuring duration for which the mice could hang from an inverted cage lid. Animals were tested for 3 minutes. A single mouse was placed on a wire lid of a standard mouse cage; lid was slightly shaken to induce gripping before inverting. Vertical distance between wire lid and bottom of the cage was 37 cm. Duration the mouse held on to the lid with at least two limbs was tested. Each animal was tested for 180 seconds per trial, for three times and average was calculated and compared [24].

## Narrow beam walk test:

Pre-trained mice were allowed to walk on a narrow horizontal stationary wooden beam (L100cm X W1cm) placed at a height of 100cm from the floor. Time taken to travel from one end of the beam to the other was recorded. Each mouse was given three trials and the average was computed and compared [25].

## Statistical analysis:

Unpaired t test was used to evaluate the effects of MPTP administration (Control mice vs. MPTP mice) and housing condition (Standard housing or Enriched housing) and on performance outcomes. p value <0.05 was considered significant.

## Results and Discussion:

Neuromuscular endurance, assessed using the Hang test, revealed a significant effect of MPTP administration in mice housed under standard conditions (t = 2.51, p = 0.05). MPTP-treated mice exhibited significantly poorer performance by falling earlier, compared to controls. (171.47+/-1.28, 176.31+/-3.62). Exposure to enriched housing significantly affected the performance of PD mice in the Hang test demonstrated by the mice gripping the lid for longer. (t = 2.50, p = 0.05). (175.67 +/- 4.08, 171.47+/-1.28) Figure 1 (see PDF). Motor coordination and balance, evaluated using the Rotarod test, showed that MPTP-administered mice in standard housing performed significantly worse than controls, by falling earlier (t = 6.91, p < 0.001) (579.03 +/- 7.43, 598.2+/-2.63). In contrast, MPTP mice housed in an enriched environment demonstrated significantly longer latency to fall on the Rotarod compared to their counterparts in standard housing (t = 5.76, p < 0.001).(591.27+/-8.83, 579.03+/-7.43) Figure 1 (see PDF). Spontaneous locomotor activity, measured using the Actophotometer, indicated that MPTP mice in standard housing displayed significantly reduced locomotion relative to control mice (t = 6.33, p < 0.001). (235.50+/-15.39, 258.33+/-18.00) Enriched housing significantly impacted the locomotor activity of MPTP mice (t = 2.71, p = 0.02). (256.17+/-10.55, 235.50+/-15.39) Figure 1 (see PDF). Narrow beam walk - motor coordination assessed by narrow beam walk test show that walking time in MPTP mice was increased as compared to controls.(t=30.7, p<.00) (7.43+/- 0.23, 4.03+/-0.15,) Enriched housing decreased the beam traverse time by MPTP mice as compared to the MPTP mice in standard housing. (t=2.8, p=.01) (7.12+/-0.41, 7.52+/-0.37) Figure 1 (see PDF).

This study aimed to examine whether environmental enrichment could mitigate motor deficits in an MPTP-induced Parkinson's disease (PD) mouse model as assessed by the different behavioural tests. The findings clearly demonstrate that enriched housing significantly enhanced neuromuscular endurance, motor coordination, and spontaneous locomotion in MPTP-treated mice compared to standard housing conditions. Behavioural assessments in the subacute MPTP model have yielded variable results across studies. While some report consistent motor impairments, others observe minimal or no deficits in tasks such as the Rotarod, Hang test, and Actophotometer [26, 27, 28- 29]. In our study, PD mice displayed clear motor deficits. Consistent with Rehman *et al.* [30] and Atiq *et al.* [31], the Hang test results showed that MPTP-treated mice had reduced grip strength and endurance, as indicated by significantly decreased hang times. Rotarod performance further validated the motor deficits in this model, aligning with findings by Ren *et al.* [32] and Zhong *et al.* [33], who reported that MPTP-treated mice exhibit impaired motor coordination and balance compared to controls evidenced by shorter time on the Rotarod. Similarly, assessment of spontaneous locomotor activity confirmed significant hypoactivity in the PD mice, consistent with previous reports by Vora *et al.* [34]. MPTP mice took a longer time to traverse the narrow beam. This is in accordance with the observation of Rai *et al.* [35]. Importantly, PD mice exposed to enriched housing demonstrated better performance across all behavioural tests, supporting the role of enrichment in mitigating neuromuscular and motor deficits. These results corroborate previous work showing that environmental enrichment can enhance muscle strength, motor endurance, and neuroplasticity in PD models [36]. Better Rotarod performance in enriched conditions further supports the neuro-protective role of enrichment, which is known to upregulate brain-derived neurotrophic factor (BDNF) and promotes dopaminergic neuron survival [37]. Significant higher scores in spontaneous locomotor activity suggests partial restoration of dopaminergic function or compensatory plasticity, consistent with earlier findings that environmental enrichment can counteract bradykinesia and hypoactivity in toxin-induced PD models [38]. Finally, decreased beam traversing time by the MPTP mice suggests that EE improves motor performance on beam walking by enhancing neuronal migration towards the lesion area in the striatum [39].

## Conclusion:

In summary, MPTP administration successfully induced Parkinsonian-like motor deficits in mice, as demonstrated by impaired performance in neuromuscular endurance, motor coordination, and locomotor activity tests. Exposure to an enriched environment significantly mitigated these deficits, highlighting the therapeutic potential of environmental enrichment as a non-pharmacological strategy to alleviate motor and behavioural impairments associated with PD. Integrating environmental enrichment with pharmacological treatments may offer an effective multimodal approach to improve functional outcomes for individuals with Parkinson's disease.
